# An Msh3 ATPase domain mutation has no effect on MMR function

**DOI:** 10.1186/s13104-017-2939-4

**Published:** 2017-11-25

**Authors:** Yasmin Edwards

**Affiliations:** Bronx Community College, 2155 University Avenue, Bronx, NY 10453 USA

## Abstract

**Objective:**

To demonstrate that the Msh3 ATPase domain is required for DNA mismatch repair and tumor suppression in a murine model.

**Results:**

The DNA mismatch repair proteins are members of the ABC family of ATPases. ATP binding and hydrolysis regulates their mismatch repair function. In the current study, a mouse model was generated harboring a glycine to aspartic acid residue change in the Walker A motif of the ATPase domain of Msh3. Impaired ATP mediated release of the *Msh2*-*Msh3* ^*GD/GD*^ complex from it’s DNA substrate in vitro confirmed the presence of an ATPase defect. However, the mismatch repair function of the protein was not significantly affected. Therefore, mutation of a critical residue within the ATPase domain of Msh3 did not preclude mismatch repair at the genomic sequences tested. Indicating that Msh3 mediated mismatch function is retained the absence of a functional ATPase domain.

**Electronic supplementary material:**

The online version of this article (10.1186/s13104-017-2939-4) contains supplementary material, which is available to authorized users.

## Introduction

DNA mismatch repair (MMR) proteins target and mediate repair of DNA polymerase errors of replication and signal the DNA damage response [1]. The MMR system consists of the highly conserved MutS and MutL homologues. In eukaryotes, the MutS homologues are Msh2, Msh3 and Msh6, which function as heterodimers. The MutSα heterodimer (Msh2-Msh6) targets single base mispairs and single base insertion/deletions for repair, and the MutSβ heterodimer (Msh2-Msh3), overlaps in the repair of single base insertion/deletions, but primarily targets larger insertion/deletions for repair [2, 3]. MutS heterodimers recruit the MutL homologues (Mlh1, Pms2, Mlh3) to mediate the next steps of repair [4]. Mutations in *MSH2, MSH6, MLH1* and *PMS2* have been identified in Hereditary non-polyposis colorectal cancer/Lynch Syndrome (HNPCC)/LS), a familial cancer syndrome [4]. Mutations in the *MSH3* gene have not been associated with Lynch Syndrome.

The ATPase domain is highly conserved and regulates the affinity of the MutS proteins for their DNA substrates [5]. Msh2 and Msh6 murine models defective in ATPase function have been generated [6, 7]. These mutations prevented ATP mediated release of the DNA substrate leading to, reduced DNA MMR, increased tumorigenesis and reduced lifespans. The impact of an Msh3 ATPase defect has not been determined in a murine model. The aim of this study was to analyze the *Msh3* ^*G855D*^ mouse line, which harbors a glycine to aspartic acid mutation in the ATPase domain of the protein, on MMR, tumor suppression and survival.

## Main text

### Methods

#### Animals

A mutation was introduced into exon 20 of the *Msh3* gene changing codon 855 from glycine (GTT) to aspartic acid (GAC) using site directed mutagenesis (Stategene, Quick change kit), following sub-cloning from a bacterial artificial chromosome. The mutation was confirmed by sequencing. A NotI fragment containing loxP sites on either side of a neomycin-PGK hygromycin resistance cassette was subcloned into an EcoRI site 150 bp upstream of exon 20. The EcoRI fragment was re-subcloned into the (+) pBluescript vector containing Msh3 genomic DNA. The vector was linearized and used to modify the Msh3 genomic locus via gene targeting in G4 embryonic stem cells (ES). Positive ES clones were identified via PCR and the correct integration confirmed using long range PCR and southern blot analysis. Positive ES cell clones were injected into C57BL6/6J females (Jackson Laboratories). One transmitted the mutant allele through its germ line. F1 males carrying the mutant allele were mated to Zp3 Cre transgenic females (C57BL/6J purchased from Jackson Laboratories), resulting in deletion of the resistance cassette by loxp mediated recombination. F1 Heterozygotes carrying the modified allele were intercrossed producing n = 20, *Msh3* ^+*/*+^, 30, *Msh3* ^*GD/*+^ and n = 22, *Msh3* ^*GD/GD*^ mice. Cohen’s effect size value of (d = 0.7). *Msh3* ^−*/*−^ animals utilized as negative controls were gifts from the laboratory of Dr. Winfried Edelmann [8]. All animals were maintained at the Albert Einstein College of Medicine animal care facility in accordance with Institutional Animal Care and Use Committee (IACUC) guidelines.

#### Electromobility shift assay (EMSA)

The effect of the *Msh3* ^*GD/GD*^ mutation on ATPase mediated DNA substrate release, was examined in nuclear extracts from each genotype. Nuclear extracts were prepared as described [9]. Nuclear extracts were prepared from pooled mouse testes obtained from five animals of each genotype *Msh2*-*Msh3* ^+*/*+^, *Msh3*-*Msh3* ^*GD/*+^, *Msh2*-*Msh3* ^*GD/GD*^ animals. Testes were minced, washed twice in cold PBS and centrifuged in 15 ml conical tubes for 4 min at 4000 rpm. Pellets were re-suspended in 5 volumes of low salt buffer (10 mM HEPES, 1.5 mM MgCl_2_, 10 mM KCl, 0.5 mM DTT and 0.5 mM PMSF), incubated on ice for 10 min, and centrifuged at 4 °C for 10 min at 4000 rpm. Pellets were re-suspended in 2 volumes of the low salt buffer, homogenized in a dounce 10–20 times and centrifuged at 4 °C for 20 min at 14,500 rpm in a Sorval. The pellet was re-suspended in high salt buffer (20 mM HEPES, 25% (v/v) glycerol, 420 mM NaCl, 1.5 mM MgCl_2_) 2 mM EDTA, 0.5 DTT, 0.5 mM PMSF,) and homogenized 3–4 times. Following homogenization, samples were incubated for 30 min at 4 °C while stirring. The mixture was centrifuged at 14,500 rpm in a Sorval at 4 °C for 20 min. The supernatant was dialyzed overnight at 4C in dialysis buffer (20 mM HEPES, 20% (v/v) glycerol, 100 mM KCL, 0.2 mM EDTA, 0.5 mM DTT, 0.5 mM PMSF). The supernatant was centrifuged at 14,500 rpm in a Sorval for 20 min at 4 °C. The nuclear extracts were aliquoted and stored at – 80 °C. To prepare binding reactions: the sense strand oligonucleotide 5′ GCTTAGGATCATCGAGGATCGAGCTCGGTGCAATTCAGCGG- CA, and antisense oligonucleotide insert 5′CCGCTGAATTGCACCGAGCTCCACACACAGATTCCTCGATGATCCTAAGC 3′ and homoduplex oligonucleotide 5′ CCGCTGAATTGCACCGAGCTCGATCCTCGATGATCCTAAGC were end labeled with infrared-700 (IR700) and annealed in 1× T4 Kinase buffer with 3× molar ratio of antisense oligonucleotide containing a 4CA insert. Thirty micrograms of nuclear extract was pre-incubated in 1× DNA binding buffer, 1ug of poly (dI–dC) and 1 ng of unlabeled homoduplex for 5 min on ice in a total volume of 19ul. Five nanograms of radio labeled DNA probe was added and the mixture incubated at room temperature in the dark for 20 min. For cold competitor reactions, the cold competitor was added in the pre-incubation reaction. ATP mediated release was induced by adding ATP 15 min following addition of DNA. The binding reaction mixture was electrophoresed on a 5% polyacrylamide gel in 0.5× trisborate EDTA (TBE) buffer. The gel was imaged using the Odyssey Infrared Imaging System (LI-COR). Image J software was use to analyze the binding intensities graphed in the binding curves.

#### Microsatellite instability analysis

Microsatellite sequences were analyzed by single cell PCR to determine the effect of Msh3^G855D^ mutation on microsatellite stability. Equal amounts of tail DNA from five mice of each genotype (*Msh3* ^+*/*+^, *Msh3* ^*GD/GD*^, and *Msh3* ^−*/*−^ *)* were pooled separately and diluted to between 0.5 and 1.0 genome equivalents. DNA was extracted from tumors and amplified for MSI analysis (not all tumors amplified). PCR cycling parameters for U12235, D17MIT91 and (TG)_27_ markers were performed as previously described [10]. A 95 °C for 1 min, 57–63° C for 1 min, and 72 °C for 1 min for 30 cycles and 72 °C for 5 min once. The products were diluted by loading buffer, heated at 95 °C for 5 min, and loaded onto 6% vertical polyacrylamide gels. Following electrophoresis, gels were fixed, dried, and exposed to X-ray film overnight (12 h) to 2 days. A single observer analyzed samples and a second observer reviewed equivocal samples.

#### Histopathological analysis of tumors

Moribund mice were identified and sacrificed using CO_2_ according to IACUC protocols. Tumors were removed and fixed in 10% neutral buffered formalin. Tumors were then embedded in paraffin and sections were removed for hematoxylin and eosin staining and analyzed.

#### Statistical analysis

The Kaplan–Meier survival curve was generated and analyzed using Graphpad prism 3.0 software. Microsoft Excel version 14.7.6 was used to determine the p values between *Msh2*-*Msh3* ^*GD/GD*^ *Msh32*-*Msh3* ^+*/*+^ and *Msh2*-*Msh3* ^−*/*−^ somatic MSI using the student t test. The Fisher exact test was used to determine the significance of tumor MSI rates. G*Power software version 3.1 was used to analyze sample size significance. Differences were determined to be statistical significant at p values < 0.05.

## Results

### *Msh2*-*Msh3*^*GD/GD*^ impaired ATP mediated DNA substrate release

**Fig. 1:**
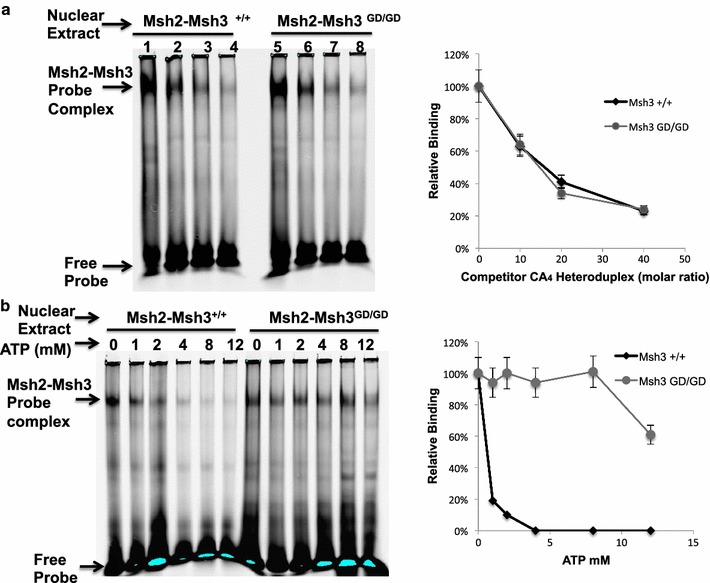
Electromobility shift assays. **a** Increasing concentrations of cold CA_4_ competitor induced substrate release by both Msh2-Msh3^+/+^ and Msh2-Msh3^GD/GD^ heterodimers. Bars indicate that there is less than 10% difference between Msh2-Msh3^GD/GD^ binding compared to wild type except at 20X molar ratio. **b** Increasing ATP concentration resulted in release of DNA binding in the Msh2-Msh3^+/+^ but not the Msh2-Msh3^GD/GD^ nuclear extracts. Percent difference bars (set at 10%) show no overlap in binding upon addition of ATP

Dissociation of the *Msh2*-*Msh3* ^+*/*+^ (positive control) and *Msh2*-*Msh3* ^*GD/GD*^ complexes from the DNA substrate was observed upon addition of increasing concentrations of cold competitor to the binding reactions, confirming the specificity of binding (Fig. 1a). The addition of increasing concentrations of ATP to the binding reactions, induced release of the *Msh2*-*Msh3* ^+*/*+^ heterodimer from the substrate, while the *Msh2*-*Msh3* ^*GD/GD*^ heterodimer persisted at the highest ATP concentrations (Fig. 1b). At physiological ATP concentrations of approximately 3–5 mM [11], the mutation reduced ATP induced DNA substrate release. Preliminary titration reactions were completed as shown in Additional file 1.

### Somatic microsatellite instability in *Msh2*-*Msh3*^*GD/GD*^ animals

Microsatellite instability is a hallmark of MMR deficiency [12]. Somatic MSI in the *Msh2*-*Msh3* ^*GD/GD*^ animals was compared to *Msh2*-*Msh3* ^+*/*+^ and *Msh2*-*Msh3* ^−*/*−^ animals (the positive and negative controls). At the TG_27_ and D7Mit91 dinucleotide markers, the highest instability was observed in the *Msh2*-*Msh3* ^−*/*−^ animals, 15 and 9% respectively, with p values of 0.03 and 0.02 compared to wild type (Table 1). Conversely, no significant instability was observed in the *Msh2*-*Msh3* ^*GD/GD*^ and *Msh2*-*Msh3* ^+*/*+^ animals, at the dinucleotide markers. Indicating that the *Msh2*-*Msh3* ^*GD/GD*^ complex retained MMR function at the dinucleotide repeat sequences tested. The *Msh2*-*Msh3* ^−*/*−^ animals showed no instability at the mononucleotide marker (Table 1). While MSI in the *Msh2*-*Msh3* ^*GD/GD*^ animals was significantly increased compared to *Msh2*-*Msh3* ^+*/*+^ animals at the U12235 mononucleotide marker (p value 0.03). At the dinucleotide sequences, the *Msh2*-*Msh3* ^*GD/GD*^ animals were similar to wild type and not the *Msh2*-*Msh3* ^−*/*−^ animals. Confirming that Msh3 MMR function remained. Tumor MSI was tested at the U12235 (A)n and TG_27_ markers (no instability was observed at the D7Mit91 marker in the tumors tested). Comparison of MSI negative tumors from wild type animals to the tumor numbers in the *Msh2*-*Msh3* ^*GD/GD*^ animals proved significant using the Fisher exact test, p value = 0.03. MSI in tumors was similar to the somatic MSI, with greater instability at the mononucleotide marker in the *Msh2*-*Msh3* ^*GD/GD*^ tumors and at the dinucleotide marker in the *Msh2*-*Msh3* ^−*/*−^ tumors. These numbers were not significant (Table 1).

**Table 1:** Somatic and tumor MSI

Marker	Msh2-*Msh3* ^+/+^	Msh2-*Msh3* ^GD/GD^	Msh2-*Msh3* ^−/−^
Somatic MSI			
U12335(A)_n_	(5/95) 5%	(16/72) 22%	(1/99) 1%
TG_27_	(3/90) 3%	(8/95) 8%	(11/75) 15%
D7Mit91	(0/77) 0%	(1/77) 1%	(7/79) 9%
Tumor MSI			
U12335	(0/6) 0%	(4/8) 50%	(2/15) 13%
TG_27_	(0/6) 0%	(1/8) 13%	(4/8) 50%

Somatic and tumor microsatellite instability: MSI was analyzed using the mononucleotide marker U12235n and dinucleotide markers TG_27_ and D7Mit91 using pooled tail DNA from five each, *Msh2*-*Msh3* ^+*/*+^ *, Msh2*-*Msh3* ^−*/*−^ *and Msh2*-*Msh3* ^*GD/GD*^ mice, (p values U12335: Msh3^+/+^ vs. Msh3^GD/GD^ = 0.036 and Msh3^+/+^: Msh3^−/−^ = 0.02, p values TG_27_: Msh3^+/+^ vs. Msh3^GD/GD^ = 0.06 and Msh3^+/+^ vs. Msh3^−/−^ = 0.038, p values D7Mit91: Msh3^+/+^ vs. Msh3^−/−^ = 0.05)

### Late stage microsatellite unstable tumors observed with no significant loss of survival

**Fig. 2:**
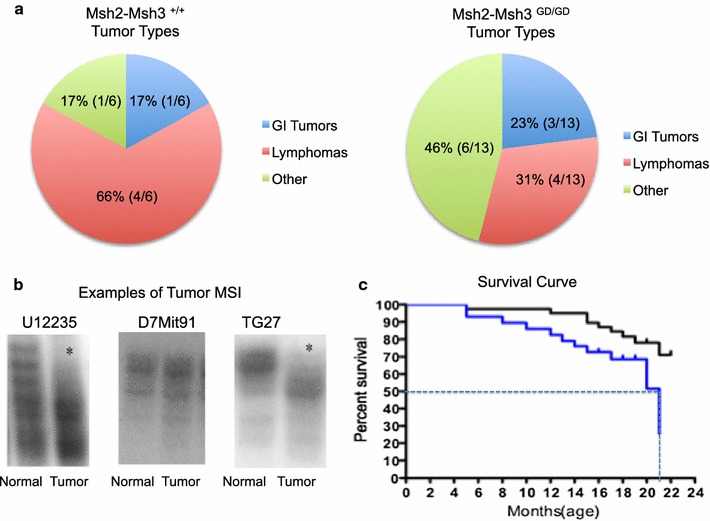
Tumor phenotype and survival curve. **a** Tumor histopathology was determined in *Msh2*-*Msh3* ^+*/*+^ and *Msh2*-*Msh3* ^*GD/GD*^ animals. Gastrointestinal tumors in the *Msh2*-*Msh3* ^+*/*+^ animals included an adenoma and the other tumors included a histiocytic sarcoma. The GI tumors in the *Msh2*-*Msh3* ^*GD/GD*^ animals included an adenoma, carcinoma and a polypoid adenomadous hyperplasia, the other tumors included two hepatic carcinomas, an extramedullary myeloma, bronchio-alveolar adenoma and two hepatic adenomas. **b** Tumor microsatellite amplifications show contractions in Msh2-Msh3^GD/GD^ tumors, no MSI was observed using the D7Mit91 marker in the tumors tested. **c** Survival curve represents the time of death or the time at which the mice became moribund and were sacrificed. The survival curves were generated using Graphpad prism 3.0 software. The black line, Msh2-Msh3^+/+^ mice (n = 20), the grey line represents the Msh2-Msh3^GD/GD^ (n = 22) animals. The 50% survival was found to be 21 months in the Msh2-Msh3^GD/GD^ animals, p value > 0.05

Analysis of tumors revealed GI tumors, lymphomas and other tumors Fig. 2a. Low tumor numbers were consistent with a weak tumor phenotype in the *Msh2*-*Msh3* ^*GD/GD*^ animals revealing no significant difference compared to *Msh2*-*Msh3* ^+*/*+^ tumorigenesis. The *Msh2*-*Msh3* ^*GD/GD*^ tumors showed both contractions and expansions (Fig. 2b). There was no significant reduction in survival between *Msh2*-*Msh3* ^*GD/GD*^ animals (21 months) compared to wild type animals (24 months), Fig. 2c. *Msh2*-*Msh3* ^*GD/GD*^ animals succumbed to tumors starting at 12th months, similar to the tumor onset previously reported in *Msh2*-*Msh3* ^−*/*−^ mice [8]. Wild type animals began to succumb later, at 16th months (Fig. 2c).

## Discussion

ADP binding in the nucleotide pocket of the Msh3 ATPase domain, is thought to increase the affinity of the Msh2-Msh3 heterodimer for DNA, while ATP binding and hydrolysis initiates recruitment of the MutL proteins and additional repair factors required to complete repair and ultimately release the substrate [13–15]. Perversely, in the current study a defective Msh3 ATPase domain did not abrogate the MMR function of the Msh2-Msh3 complex. One study, suggested that the MSH3 subunit of the heterodimer was most involved in targeting and binding of the insertion substrate, while ATP binding in the MSH2 subunit initiated the recruitment of the MutL proteins to begin the final steps of repair and release [13]. As this model would predict, the *Msh2*-*Msh3* ^*GD/GD*^ complex bound to the DNA substrate, confirming that DNA binding was not impaired by the mutation. While a defect in ATP induced release of the CA_4_ substrate was confirmed. The EMSA binding reactions were conducted in vitro and the timeline of the binding stoichiometry was not explored. Repair at dinucleotide sequences confirmed Msh3 MMR function, notwithstanding the inability to hydrolyze ATP in the Msh3^GD/GD^ subunit. A functional Msh2 ATPase domain appeared sufficient to promote delayed release from the DNA substrate, facilitating the final steps of repair in vivo.

MMR function was not significantly impaired in the *Msh3* ^*G855D*^ mouse line. The mutation led to increased instability at the genomic mononucleotide repeat sequence, and in the tumors tested. Some *Msh2*-*Msh3* ^*GD/GD*^ animals succumbed to these tumors at earlier ages than did wild type animals, but the survival rates were not significantly reduced. The results suggest that Msh3 is not a primary driver of tumorigenesis. The *Msh2*-*Msh3* ^*GD/GD*^ phenotype shows greater similarity to late onset sporadic tumorigenesis and not the familial Lynch syndrome with which mutations in the MSH2 and MSH6 MutS homologues are associated [16].

## Limitations

- ATP was added in the final 5 min of the binding reactions and so the effect of lengthier binding reaction times on DNA substrate release dynamics in the presence of ATP was not evaluated.
- The inclusion of additional dinucleotide markers, in addition to trinucleotide and tetranucleotide genomic repeat markers would be more informative in light of Msh3′s role in maintaining stability at these sequences.

## Additional file

**Additional file 1.** Preliminary electromobility shift assay (EMSA)—Preliminary EMSA including negative control (lane 1) and fixed concentrations of cold competitor (30×) in lanes 5, 6 and 7. ATP (4 mM) with Msh2-Msh3^+/+^, Msh2-Msh3^GD/+^ and Msh2-Msh3^GD/GD^ nuclear extracts in lanes 8, 9 and 10. Dissociation of DNA protein complexes was incomplete indicating that concentration of DNA and competitors needed to be adjusted.

## Abbreviations

HNPCC: Hereditary non-polyposis colorectal cancer
LS: Lynch syndrome
Msh3: MutS homologue 3
MMR: mismatch repair
MSI: microsatellite instability
