# Supplementary figures and images for: An Msh3 ATPase domain mutation has no effect on MMR function
Source: BMC Res Notes. 2017 Nov 25;10:616. doi: 10.1186/s13104-017-2939-4 (PMC5702223; doi:10.1186/s13104-017-2939-4)

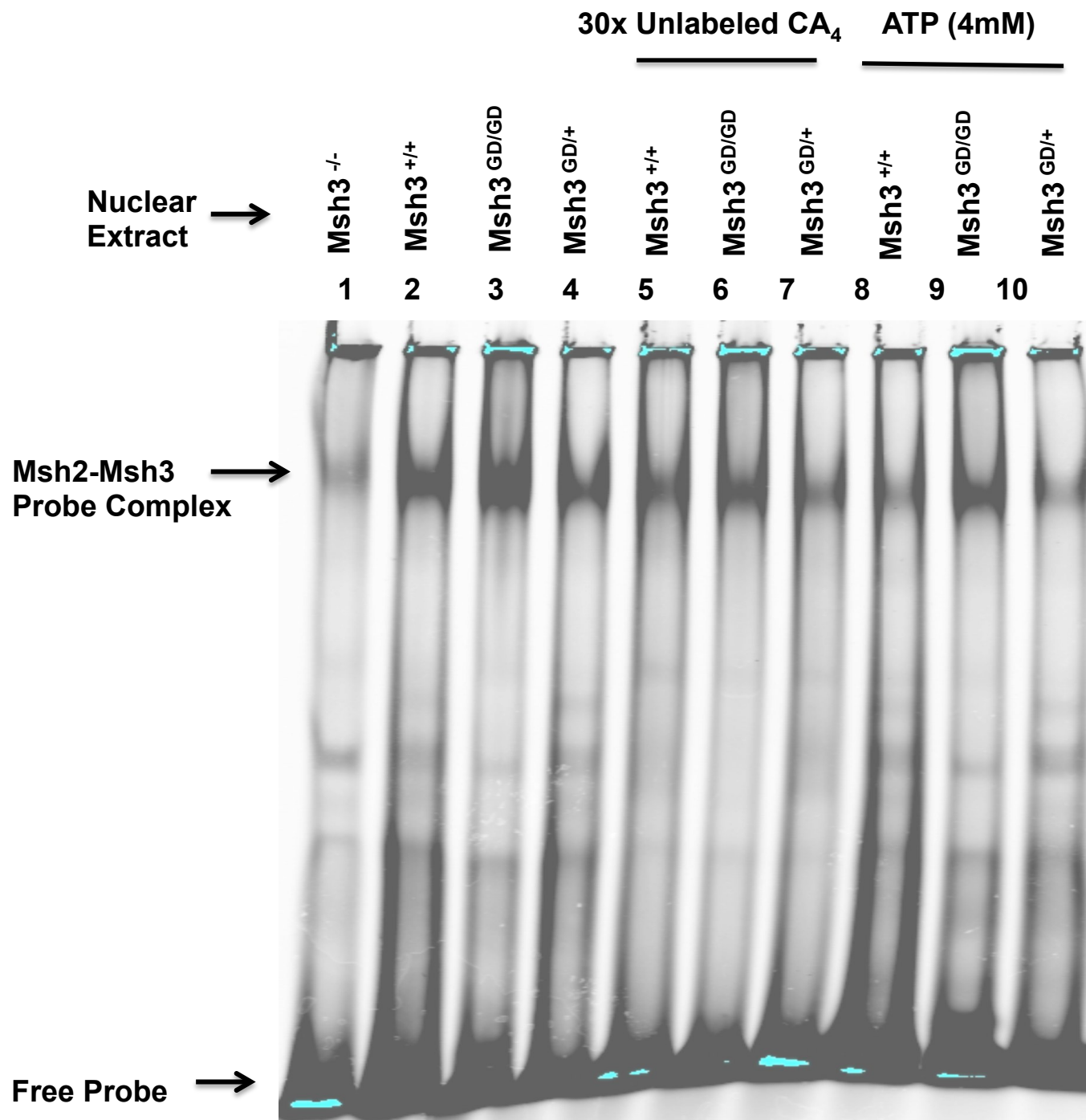

Supplement: Supplementary file 1 — Additional file 1. Preliminary electromobility shift assay (EMSA)—Preliminary EMSA including negative control (lane 1) and fixed concentrations of cold competitor (30×) in lanes 5, 6 and 7. ATP (4 mM) with Msh2-Msh3+/+, Msh2-Msh3GD/+ and Msh2-Msh3GD/GD nuclear extracts in lanes 8, 9 and 10. Dissociation of DNA protein complexes was incomplete indicating that concentration of DNA and competitors needed to be adjusted. [file 13104_2017_2939_MOESM1_ESM.pdf]
